# Evaluation of the Ability of Miltefosine Associated with Topical GM-CSF in Modulating the Immune Response of Patients with Cutaneous Leishmaniasis

**DOI:** 10.1155/2020/2789859

**Published:** 2020-08-06

**Authors:** Fábio Peixoto, Maurício T. Nascimento, Rúbia Costa, Juliana Silva, Gaby Renard, Luiz Henrique Guimarães, Gerson Penna, Manoel Barral-Netto, Lucas P. Carvalho, Paulo R. L. Machado, Edgar M. Carvalho

**Affiliations:** ^1^Instituto Gonçalo Moniz, FIOCRUZ, R. Waldemar Falcão, 121, Candeal, 40296-710 Salvador, BA, Brazil; ^2^Serviço de Imunologia, Hospital Universitário Professor Edgard Santos, Universidade Federal da Bahia, R. Dr. Augusto Viana, s/n, Canela, 40301-155 Salvador, BA, Brazil; ^3^Quatro G Pesquisa & Desenvolvimento Ltda, Av. Ipiranga 6681, Prédio 92A, Porto Alegre 90619-900, Brazil; ^4^Universidade Federal do Sul da Bahia, Praça Joana Angélica, 58, São Jose, Teixeira de Freitas, 45988 BA, Brazil; ^5^Universidade de Brasília (UnB), Núcleo de Medicina Tropical, Brasília, 70910-900 DF, Brazil; ^6^Instituto Nacional de Ciência e Tecnologia em Doenças Tropicais (INCT-DT), Salvador, BA, Brazil

## Abstract

Cutaneous leishmaniasis (CL) due to *L. braziliensis* is associated with an exaggerated inflammatory response and tissue damage. Miltefosine is more effective than pentavalent antimony (Sb^v^) in the treatment of CL, and here, we evaluate the ability of Sb^v^, miltefosine, and GM-CSF administered intravenously, orally, or topically, respectively, to modify the immune response. Patients were treated with miltefosine *plus* GM-CSF, miltefosine *plus* placebo, or Sb^v^. Mononuclear cells were stimulated with soluble *Leishmania* antigen (SLA) on day 0 and day 15 of therapy, and cytokine levels were determined in supernatants by ELISA. The lymphocyte proliferation and oxidative burst were evaluated by flow cytometry, and the degree of infection and *Leishmania* killing by optical microscopy. Proliferation of CD4^+^ T cells were enhanced in patients using miltefosine and in CD8^+^ T cells when GM-CSF was associated. Enhancement in the oxidative burst occurred in the miltefosine *plus* GM-CSF group on day 15 of therapy. Moreover, the number of *L. braziliensis* in infected monocytes on day 15 as well as the percentage of infected cells was lower after 48- and 72-hour culture in cells from patients treated with miltefosine *plus* GM-CSF. In addition to the ability of miltefosine to kill *Leishmania*, the changes in the immune response caused by miltefosine and GM-CSF may increase the cure rate of CL patients using these drugs.

## 1. Introduction

Tegumentary leishmaniasis (TL), caused by the protozoan of the genus *Leishmania*, is a major health problem in many regions of the world. After being transmitted by sand flies, *Leishmania* parasites infect human macrophages and dendritic cells (DCs), causing a wide spectrum of clinical manifestations, including visceral leishmaniasis (VL), cutaneous leishmaniasis (CL), disseminated leishmaniasis (DL), mucosal leishmaniasis (ML), and diffuse cutaneous leishmaniasis (DCL). Cutaneous leishmaniasis (CL) in Latin America is predominantly caused by *Leishmania* (*Viannia*) *braziliensis* and is characterized by the presence of one or a few well-delimited ulcerated lesions with granulomatous fundus and elevated borders [1]. Host immunological factors play an important role in the pathogenesis of the disease. Mononuclear cells from patients with CL stimulated with soluble *Leishmania* antigens (SLA) display an exacerbated Th1-type immune response and produce high levels of IFN-*γ* and TNF and low levels of IL-10 in cultures [2]. The production of IFN-*γ* is important to prevent parasite proliferation in mononuclear phagocytes and the dissemination of the infection [3, 4]. However, this response is not capable of eliminating all the parasites, and the persistent stimulation of the immune system by the parasites and *Leishmania* antigens leads to an exaggerated inflammatory response resulting in tissue damage [5]. Furthermore, studies have shown a pathogenic role of CD8^+^ T cells at the lesion site, as the lyses of infected cells release molecules that induce the secretion of IL-1*β*, TNF, inflammasome activation, and the appearance of the ulcer [6]. In this context, inflammatory cytokines may have an important role in parasite eradication, but its overproduction is associated with tissue damage and development of the cutaneous ulcer.

Meglumine antimoniate (Sb^v^) is the first-choice drug for the treatment of CL in Latin America, but an increase in therapeutic failure, hitting over 50% of the patients, has been observed in the last 15 years [7, 8]. Other alternative drugs like pentamidine and amphotericin B have shown toxic activity to patients, and the last one requires hospitalization; therefore, their use is limited. Miltefosine is an oral leishmanicidal drug that can eliminate up to 95% of *L. donovani* and *L. infantum* amastigotes in mice [9]. Miltefosine has been effective in the treatment of CL caused by *L. braziliensis* and *L. guyanensis* [7, 10]. In Brazil, miltefosine cured 75% of patients infected with *L. braziliensis* and 71.4% of patients infected with *L. guyanensis* [7, 10].

Although miltefosine is a leishmanicidal drug, its mechanisms of action are not fully understood. It is known that miltefosine acts not only by blocking cytochrome C oxidase leading to changes in mitochondrial membrane potential and consequently reducing oxygen consumption and ATP levels in *L. donovani* [11] but also by inhibiting phosphatidylethanolamine N-methyl-transferase and therefore the biosynthesis of phosphatidylcholine [12]. Some studies also demonstrate that miltefosine opens the sphingosine-activated plasma membrane Ca^2+^ channel and has a direct effect on acidocalcisomes, which in combination result in large intracellular Ca^2+^ accumulation [13]. Furthermore, miltefosine causes an apoptosis-like death in *L. donovani* promastigotes [14].

The host immune response has a great impact and influence on the therapeutic response of CL. Patients with diffuse CL, a disease caused by *L. amazonensis* in Latin America, have a poor Th1-type immune response and are refractory to therapy [15]. Alternatively, the presence of an exaggerated immune response, as observed in CL caused by *L. braziliensis*, does not prevent the development of the disease [16, 17]. Previous studies showed that Sb^v^ associated with granulocyte and macrophage colony stimulation factor (GM-CSF) or pentoxifylline (a drug that decreases TNF production) is more effective and reduces the healing time of CL and ML [18–21]. GM-CSF induces *in vitro* macrophage activation and increases *Leishmania* killing [22–24]. Miltefosine has the ability not only to kill *Leishmania* but also to enhance chemotaxis, motility, monocyte adhesion, and phagocytosis [25]. Studies indicate that the ability of miltefosine to stimulate macrophage and monocyte activation is due to its semblance with phosphatidylcholine, enhancing the membrane fluidness of these cells [25]. The aim of this study was to determine the changes in the immune response in patients treated with intravenous antimony, oral miltefosine, and topical GM-CSF.

## 2. Materials and Methods

### 2.1. Patients

Participants of this study were patients with CL from the endemic area of Corte de Pedra, Bahia, Brazil, who were participating in an ongoing randomized control study aimed at comparing the efficacy and effectivity of miltefosine associated to GM-CSF versus miltefosine *plus* placebo and antimoniate of meglumine. The diagnosis of CL was performed by the presence of a typical CL ulcer and detection of DNA of *L. braziliensis* by PCR. Patients were allocated into three groups. Patients in group 1 received Impavido™ (Paesel + Lorei, Rheinberg, Germany) at a dose of 2.5 mg/kg/d for 28 days with a maximum dose of 150 mg/day orally + topical GM-CSF (gel cream 0.01%, twice a day per 28 days). Patients in group 2 were treated with miltefosine (Impavido) using the same dose and schedule as group 1 + topical placebo (gel cream twice a day per 28 days). Patients in group 3 received Glucantime (Sanofi-Aventis) at a dose of 20 mg/kg/d intravenously for 20 days with a maximum dose of 1200 mg. The blood samples of these individuals were collected before treatment (day 0) and during therapy on day 15. Inclusion criteria were age between 18 and 60 years old, illness duration of more than 20 and less than 90 days, and size of the ulcer between 10 and 40 mm.

### 2.2. Ethical Statement

All patients agreed to participate in the study and signed their informed consent. This study was approved by the Institution Review Board of the Federal University of Bahia Medical School under the number 55647116.2.0000.5577.

### 2.3. Immunological Studies

#### 2.3.1. Separation of Peripheral Blood Mononuclear Cells

Peripheral blood mononuclear cells (PBMC) were obtained from heparinized venous blood by density gradient centrifugation using Ficoll-Hypaque (GE Healthcare). Cells were washed twice in saline and were resuspended at the desired concentration in RPMI 1640 supplemented with 10% bovine fetal serum (both produced by Gibco, Grand Island, NY, USA) plus antibiotics (complete RPMI).

#### 2.3.2. Cytokine Production Determination

PBMC were adjusted to 3 × 10^6^/mL in complete RPMI and cultured in a 37°C, CO_2_ incubator for 72 hours in the presence or absence of SLA (5 *μ*g/mL). The supernatants of those cultures were collected and utilized for the measurement of Granzyme B, IL-1*β*, IL-10, IFN-*γ*, TNF, and the chemokines CXCL9 and CXCL10, through ELISA sandwich as previously described.

#### 2.3.3. Lymphocyte Proliferation Essay

To evaluate lymphocyte proliferation, 1 × 10^6^ PBMC were cultured in the presence or absence of SLA (5 *μ*g/mL). After 5 days of incubation at 37°C with 5% CO_2_, cells were marked with the conjugated antibodies anti-CD4 and anti-CD8, with the goal of separating the lymphocyte subpopulations, and anti-Ki-67 as a cell proliferation marker. Afterwards, cells were analyzed by flow cytometry and data were analyzed by FlowJo®. Cells stimulated with *α*CD3 + *α*CD28 were the positive control.

#### 2.3.4. Oxidative Burst Quantification

To evaluate the reactive oxygen species, 1 × 10^6^ PBMC were treated with dihydrorhodamine-123 at 10 ng/mL (Cayman Chemical Company) for 10 minutes. After that, cells were infected with *Leishmania braziliensis* (Lb), 5 *Leishmania* parasites per monocyte, considering that this cell type is 15% of the total PBMC, for 25 minutes, and then stained with anti-HLA-DR and anti-CD14 fluorochrome-conjugated antibodies. The fluorescence intensity of the cells was assessed by flow cytometry, and data were analyzed through FlowJo®. Positive controls were cells stimulated with phorbol myristate acetate.

#### 2.3.5. Infection with *L. braziliensis*

To evaluate the infection rate, 2.5 × 10^6^ cells/mL were incubated in Nunc® Lab-Tek® plates for 2 hours for monocyte adhesion, and the nonadherent cells were washed out of the plate. Adherent cells (90% monocytes) were infected with *L. braziliensis* (5 parasites per monocyte) for two hours, and the remaining promastigotes were washed out of the plate. Cells were cultured for 2, 48, or 72 hours at 37°C with 5% CO_2_. The slides were stained with panoptic stain for quantification of infected monocytes and the number of amastigotes per 100 monocytes was determined by optical microscopy.

### 2.4. Statistical Analysis

Mann-Whitney's test was used for comparisons between 2 independent continuous variables, Wilcoxon's *U* test was used for continuous dependent variables, and the Kruskal-Wallis's test and Dunn's posttest were used to compare 3 continuous variables. *p* < 0.05 was considered statistically significant, and all *p* values represented are 2-tailed. All experiments were statically analyzed through Prism GraphPad® 8.0.2, such as the graphics elaboration.

## 3. Results

### 3.1. Cytokine Production

**Figure 1:**
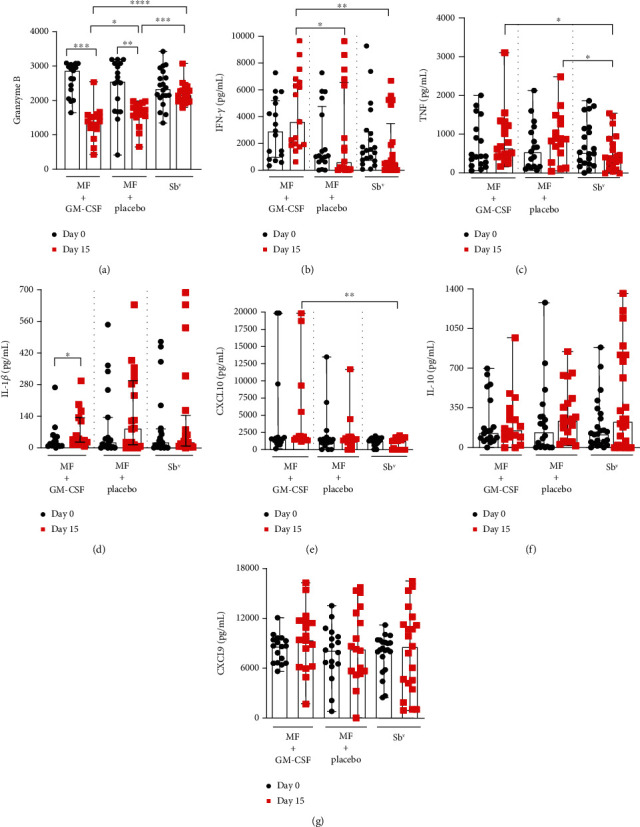
Cytokine production by PBMC from CL patients during therapy. PBMC from patients treated with miltefosine + GM-CSF (*n* = 17), miltefosine + placebo (*n* = 17) and Sb^v^ (*n* = 21) were stimulated with SLA (5 *μ*g/mL) for 72 hours on day 0 and 15 of therapy. (a) Granzyme B, (b) IFN-*γ*, (c) TNF, (d) IL-1*β*, (e) CXCL10, (f) IL-10, and (g) CXCL9 levels were determined in culture supernatants by ELISA. The data is represented with median and interquartile range. Statistical analyses were performed using Wilcoxon's or Mann-Whitney's rank test; ^∗^*p* < 0.05, ^∗∗^*p* < 0.01, ^∗∗∗^*p* < 0.001, and ^∗∗∗∗^*p* < 0.0001.

PBMC from CL patients treated with miltefosine *plus* GM-CSF, miltefosine *plus* placebo, or Sb^v^, were stimulated with SLA before and on day 15 of therapy, and the production of Granzyme B, IFN-*γ*, TNF, IL-1*β*, IL-10, CXCL9, and CXCL10 were compared among the groups. Granzyme B production (Figure 1(a)) in patients treated with miltefosine *plus* GM-CSF decreased from 2888 pg/mL (1654-3111 pg/mL) in day 0 to 1401 (436-2564 pg/mL) in day 15, (*p* = 0.0001). The same effect was observed in miltefosine *plus* placebo-treated patients in which Granzyme B production decreased from 2574 pg/mL (418-3219 pg/mL) in day 0 to 1657 (650.2-1998 pg/mL) on day 15 (*p* = 0.0021). When comparing groups among each other during treatment (day 15), we could observe lower levels of this protein in the miltefosine *plus* GM-CSF group than in the miltefosine *plus* placebo (*p* = 0.01) and meglumine antimoniate groups, 2183 pg/mL (1810-3102 pg/mL) (*p* < 0.0001). Regarding IFN-*γ* (Figure 1(b)), patients treated with miltefosine *plus* GM-CSF presented a higher level on day 15 of therapy, 1627 pg/mL (162-6351 pg/mL), than patients who received miltefosine *plus* placebo, 433.5 pg/mL (0-9739 pg/mL) (*p* = 0.048), or Sb^v^, 239 pg/mL (0-742 pg/mL) (*p* = 0.0032). The TNF level (Figure 1(c)) observed in patients inserted in the same treatment group (MF *plus* GM-CSF) was 654 pg/mL (244-3127 pg/mL), and the TNF level in patients treated with miltefosine *plus* placebo was 879 pg/mL (54-2500 pg/mL), which were higher than those found in patients treated with Sb^v^, 382 pg/mL (0-1550 pg/mL) (*p* = 0.03 and *p* = 0.046). The IL-1*β* production (Figure 1(d)) in patients treated with miltefosine *plus* GM-CSF increased from 38 pg/mL (8-92 pg/mL) on day 0 to 128 pg/mL (26-300 pg/mL) (*p* = 0.01). Neither of the other treatment options modified this cytokine production. The level of CXCL10 (Figure 1(e)) was higher in the miltefosine *plus* GM-CSF group, 1687 pg/mL (1177-20000 pg/mL), than in the Sb^v^ group, 1384.5 pg/mL (15-2087 pg/mL) (*p* = 0.03). Regarding IL-10 and CXCL9, no statistical difference has been observed.

### 3.2. Lymphocyte Proliferation

**Figure 2:**
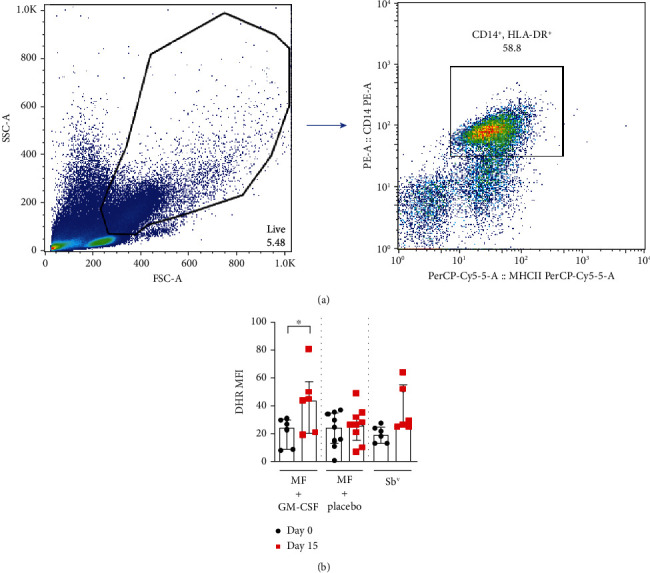
Miltefosine *plus* GM-CSF treatment enhances reactive oxygen species production by monocytes after *L. braziliensis* infection. Monocytes from CL patients were evaluated on day 0 and 15 of treatment with miltefosine + GM-CSF (*n* = 6), miltefosine + placebo (*n* = 9), and Sb^v^ (*n* = 6). The cells were treated with DHR (10 ng/mL—10 min) and infected with *L. braziliensis* promastigotes for 25 minutes at a 5 : 1 ratio. Cells were stained with anti-CD14 and anti-HLA-DR. Data were collected using flow cytometry and analyzed using FlowJo® software. (a) Representative gating strategy on CD14^+^ and HLA-DR^+^ expression in monocytes from one CL patient. DHR MFI was taken from CD14^+^ HLA-DR^+^ population. (b) The data represent the mean of fluorescence intensity (MFI) of oxidative burst production by monocytes from CL patients inserted in the treatment groups. The data is represented with median and interquartile ranges. Statistical analyses were performed using Mann-Whitney's test for unpaired groups and Wilcoxon's rank test for paired measurements; ^∗^*p* < 0.05.

PBMC were cultured for 5 days for the evaluation of CD4^+^ and CD8^+^ T cell proliferation through Ki-67 expression. To accomplish that, we quantified the frequency of T cells expressing this molecule in day 15 and divided this by the frequency found in day 0, allowing the analysis of drug capacity in inducing proliferation. Using this index, we observed an increase in CD4^+^ T proliferation in patients treated with miltefosine *plus* GM-CSF and miltefosine *plus* placebo, whereas a decrease in the Sb^v^ group was noted. The same procedure was done with CD8^+^ T cells and, with miltefosine *plus* GM-CSF as an exception, a decrease in proliferation was observed during the treatment (Figure 2(b)).

### 3.3. Oxidative Burst

**Figure 3:**
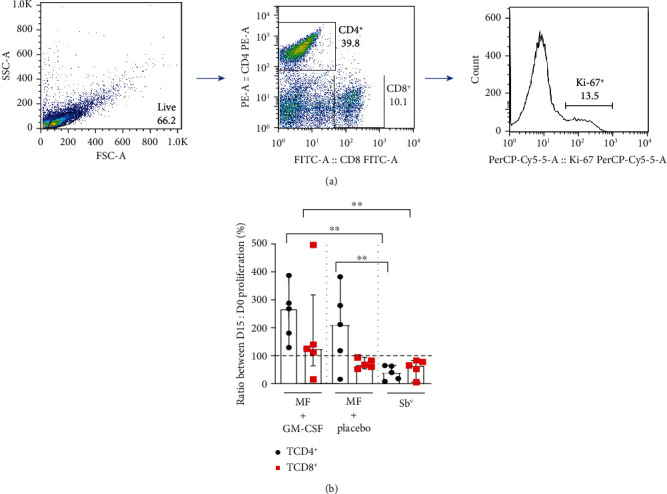
Miltefosine treatment induces CD4^+^ T cell proliferation, and its association with GM-CSF enhances CD8^+^ T cell proliferation by PBMC from CL patients. PBMC from CL patients treated with miltefosine + GM-CSF (*n* = 5), miltefosine + placebo (*n* = 5), and Sb^v^ (*n* = 5) were cultured for 5 days in the presence of SLA on day 0 and 15 of therapy. Cells were stained with anti-CD4, anti-CD8, and anti-Ki67. Data were collected using flow cytometry and analyzed by FlowJo® software. (a) Representative gating strategy on CD4^+^, CD8^+^, and Ki-67^+^ expression in lymphocytes from one CL patient. (b) The data represent the ratio between the proliferation found at day 15 and day 0 of treatment from CL patients inserted in the treatment groups added by 100. The data is represented with median and interquartile range. Statistical analyses were performed using Mann-Whitney's test for unpaired groups and Wilcoxon's rank test for paired measurements; ^∗^*p* < 0.05 and ^∗∗^*p* < 0.01.

The reactive oxygen species (ROS) produced by monocytes from CL patients after 25 minutes of infection with *L. braziliensis* before and on day 15 of therapy is shown in Figure 3. The median fluorescence index (MFI) of DHR 123 of monocytes from patients treated with miltefosine *plus* GM-CSF enhanced from 25 (8-31) to 44.5 (19-81) from day 0 to day 15 (*p* = 0.03) (Figure 3(b)). There was no difference in the oxidative burst during therapy in the other groups.

### 3.4. Infection Rate and Parasite Killing

**Figure 4:**
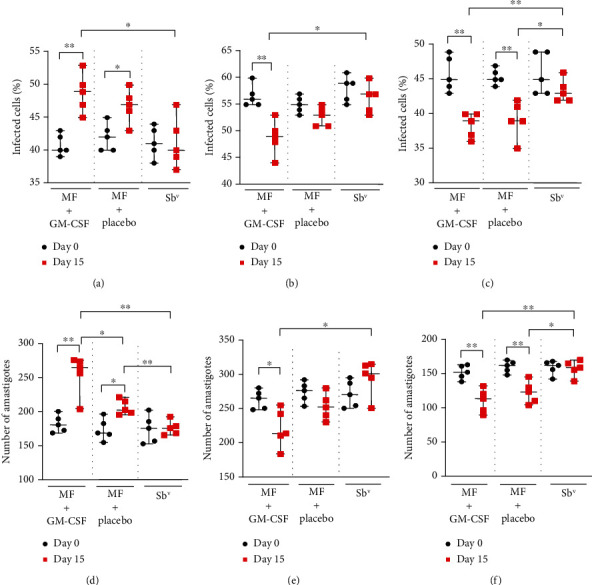
Influence of miltefosine and GM-CSF treatment in the phagocytosis and killing of *L. braziliensis* by monocytes from CL patients. Monocytes from CL patients treated with miltefosine + GM-CSF (*n* = 5), miltefosine + placebo (*n* = 5), and Sb^v^ (*n* = 5) were infected with *L. braziliensis* promastigotes at a 5 : 1 ratio for 2, 48, and 72 hours. The percentage of infected cells after 2 hours (a), 48 hours (b), and 72 hours (c) as well as the number of intracellular parasites after 2 (d), 48 (e), and 72 (f) hours were determined by optical microscopy, after panoptic staining on day 0 and day 15 of therapy. The data is represented with median and interquartile range. Statistical analyses were performed using Mann-Whitney's test for unpaired groups and Wilcoxon's rank test for paired measurements; ^∗^*p* < 0.05, ^∗∗^*p* < 0.01.

Monocytes were isolated through adhesion in Nunc® Lab-Tek® chambers, infected with *L. braziliensis*, and cultured for 2, 48, and 72 hours. After 2 hours of incubation, we observed that monocytes from miltefosine *plus* GM-CSF and miltefosine *plus* placebo-treated patients had higher infection rate on day 15 of therapy than before treatment, 49 (45-53) versus 40 (39-43) (*p* = 0.0079) and 47 (43-50) versus 42 (40-45) (*p* = 0.02) (Figure 4(a)), as well as a higher number of amastigotes internalized per 100 monocytes, 266 (205-277) versus 181 (169-201) (*p* = 0.008) and 203 (196-222) versus 169 (155-197) (*p* = 0.015) (Figure 4(d)). When monocytes were obtained on day 15 and were cultured for 48 hours, we observed a decrease in the infection ratio in the miltefosine *plus* GM-CSF group, 49 (44-53) versus 56 (55-60) at day 0 (*p* = 0.03) (Figure 4(b)), and a decrease after 72 hours by both the miltefosine *plus* GM-CSF-treated group, 39 (36/40) in day 15 versus 45 (43/49) in day 0 (*p* = 0.008), and the miltefosine *plus* placebo-treated group (*p* = 0.009) (Figure 4(c)). The same phenomena were observed when comparing the number of amastigotes during versus before therapy. At day 15 of therapy, when monocytes were cultured for 48 hours, there was a decrease in parasites internalized by monocytes in the miltefosine *plus* GM-CSF group (*p* = 0.03) (Figure 4(e)); when monocytes were cultured for 72 hours, there was a similar decrease in internalized parasites in the miltefosine *plus* GM-CSF-treated group from 153 (139-162) to 114 (90-133) (*p* = 0.008). The same was observed in patients treated with miltefosine *plus* placebo (*p* = 0.008) (Figure 4(f)). When comparing groups, we observed a higher percentage of infected cells in the miltefosine *plus* GM-CSF group compared to antimony, 49 (45-53) versus 40 (37-47) (*p* = 0.02) (Figure 4(a)). We also observed a higher number of amastigotes internalized by monocytes from the first group of patients, 266 (205-277), compared to the miltefosine *plus* placebo group (*p* = 0.03) and the Sb^v^ group, 176 (166-193) (*p* = 0.007), after 2 hours of incubation (Figure 4(d)). The miltefosine *plus* placebo group also presented higher levels of amastigote internalization than meglumine antimoniate-treated patients (*p* = 0.008) (Figure 4(d)). After 48 hours, the relation between the miltefosine *plus* GM-CSF group and the antimony group is inverted, and patients from the second group presented a higher percentage of infected cells (*p* = 0.016) (Figure 4(b)) as well as a higher number of amastigotes per 100 monocytes (*p* = 0.015) (Figure 4(e)). Analyzing 72-hour cultures, we saw a lower infection ratio in both the miltefosine *plus* GM-CSF group, 39 (36-40), and the miltefosine *plus* placebo group, 39 (35-42), compared to antimony-treated patients, 43 (42-46) (*p* = 0.007 and *p* = 0.02) (Figure 4(c)), as well as lower number of amastigotes internalized, 114 (90-133) and 124 (105-146) versus 160 (140-171) (*p* = 0.007and*p* = 0.016), respectively (Figure 4(f)).

## 4. Discussion

Miltefosine is effective against visceral and cutaneous leishmaniasis, and the cure rate of this drug is higher than that observed with meglumine antimoniate in ATL (American Tegumentary Leishmaniasis). Miltefosine also has immunomodulatory properties as it increases phagocytosis and enhances IFN-*γ* production, the main cytokine that activates macrophages for *Leishmania* killing [7, 26–28]. GM-CSF has a wide effectivity on monocytes and macrophages, activating these cells and granting a leishmanicidal effect [22–24]. Furthermore, in mice infected with *Mycobacterium tuberculosis*, this molecule inducts the recruitment of macrophages and lymphocytes to the lesion site [29]. We have previously shown that GM-CSF associated to Sb^v^ increases the cure rate and reduces the healing time of cutaneous leishmaniasis [19]. In the present study, taking advantage of an ongoing clinical trial evaluating the efficacy of miltefosine *plus* GM-CSF vs. miltefosine *plus* placebo vs. Sb^v^, we compared the immunological response of CL patients before and during therapy. We observed that, *in vitro*, monocytes from patients using miltefosine *plus* GM-CSF increased the respiratory burst and decreased the percentage of infected cells as well as the number of amastigotes per 100 monocytes during treatment. Moreover, there was an increase in the percentage of CD4^+^ and CD8^+^ T cell proliferation, an increase in IL-1*β* production, and a decrease of Granzyme B concentration.

The control of *Leishmania* infection is associated with a Th1 immune response. CD4^+^ T cell activation and, consequently, IFN-*γ* production participate in host protection, and in this study, we showed that miltefosine increases the number of CD4^+^ T cells, which may favor parasite killing and release of *Leishmania* antigens and, consequently, more lymphocyte activation. In addition to an increase in CD4^+^ T cells, miltefosine therapy kept the enhancement of IFN-*γ* and TNF that are cytokines already enhanced during *L. braziliensis* infection.

Previous studies showed that IFN-*γ* is necessary for the control of parasite proliferation, granting leishmanicidal effects by mononuclear phagocytes [3, 4]. Thus, it is possible that, in addition of the leishmanicidal effect of miltefosine, the maintenance of IFN-*γ* levels may also contribute to parasite killing and a higher cure rate in patients treated with miltefosine *plus* GM-CSF and miltefosine *plus* placebo than in those treated with meglumine antimoniate [6, 8].

In addition to IFN-*γ* and TNF, other cytokines are highly produced during CL and some of them have been associated with the pathogenesis of the disease. CXCL9 and CXCL10 are produced in high levels during *L. braziliensis* infection and are associated with the chemotaxis of T and NK cells to the lesion site [30]. Alternatively, IL-10, a regulatory cytokine, may favor parasite proliferation but may attenuate tissue damage during *Leishmania* infection [31]. However, we did not observe any important influence of the drugs used in modifying the production of these molecules.

Recently, emphasis has been given to the role of cytotoxic CD8^+^ T cells and IL-1*β* production in the pathology of CL. CD8^+^ T cells from CL patients display a higher expression of Granzyme B, and while they kill *Leishmania*-infected cells, they have a limited ability to kill *L. braziliensis* [32–34]. Moreover, it has been suggested that cell killing activates inflammasome and IL-1*β* production and this inflammatory response leads to tissue damage and ulcer development [33, 35]. Here, we showed that miltefosine decreased Granzyme B production which may have contributed to a decreased pathology. Patients who received miltefosine, as well as Sb^v^, did not modify IL-1*β* production, but those treated with miltefosine plus GM-CSF had enhanced IL-1*β* levels. However, while cytotoxicity mediated by CD8^+^ T cells and Granzyme B have been associated with pathology in CL, IL-1*β* seems to have a dual effect as this cytokine may have a protective role in the early phase of the infection in mice [36].

Previous studies have shown that miltefosine changes the membrane fluidity of monocytes and macrophages, which might enhance phagocytic function by these cells [25]. When those cells were stimulated *in vitro* with miltefosine and cultured with *Saccharomyces cerevisiae*, the drug enhanced phagocytosis by macrophages as well as the number of cells engaged in this activity [27]. Here, we show for the first time that miltefosine enhances the phagocytosis of *L. braziliensis*, and more importantly, we documented a decrease in the frequency of infected cells as well as in the number of amastigotes after 48 and 72 hours of culture of monocytes from patients treated with miltefosine when compared to monocytes before treatment and also with cells from patients treated with meglumine antimoniate. *Leishmania* killing by macrophages is dependent on the respiratory burst with an increase in the production of reactive oxygen species [37]. During CL by *L. braziliensis* infection, the killing is predominantly performed by classic macrophages [35]. While in the mouse model of CL, NO production has been associated with *Leishmania* killing, in humans, we found no leishmanicidal role for this molecule. Instead, inhibition of ROS production decreased the ability of human monocytes to kill *Leishmania* [37]. Our observation that miltefosine enhances the oxidative burst indicates that in addition of inducing phagocytosis, the drug increases parasite killing.

## 5. Conclusions

Our results confirm that miltefosine enhances monocytic function and also show that this drug enhances IL-1*β* production and maintains the levels of IFN-*γ* and TNF observed before therapy. We also showed that topical use of GM-CSF associated with oral miltefosine modifies the systemic immune response increasing IFN-*γ* and ROS production and decreasing Granzyme B levels. This observation may contribute to better parasite control and an increase in the cure rate of patients with cutaneous leishmaniasis treated with miltefosine.

## Data Availability

The data used to support the findings of this study have been deposited in the figshare repository (DOI 10.6084/m9.figshare.12003192) and are included within the article.
